# Cyber Military Operations under International Humanitarian Law: Interpreting the Concept of “Attack” and Challenges in Protecting Civilians

**DOI:** 10.12688/f1000research.182654.1

**Published:** 2026-06-12

**Authors:** Eman Hamdan, Ashraf Al-Rai, Maya Khater

**Affiliations:** 1 PH-D, Collage of Law, University of Geneva, Geneva, Switzerland, 1201, Switzerland; 2 Collage of Law, Abu Dhabi University, Abu Dhabi, Abu Dhabi, 00000, United Arab Emirates; 3 Collage of Law, United Arab Emirates University, Al Ain, Abu Dhabi, 00000, United Arab Emirates

**Keywords:** Protection of civilians, cyberattacks, cyber military operations, discrimination, cyberspace.

## Abstract

**Background:**

Given the growing use of information and communication technology across many essential industries, threats associated with military operations have emerged in cyberspace, thereby resulting in various debates concerning the extent to which International Humanitarian Law applies to such threats.

**Methods:**

The paper adopts an analytical and inductive approach based on traditional and customary IHL provisions, as well as reports from specialized organizations. In this regard, this study explores the legal framework for these threats and assesses the applicability of the IHL provisions to operations carried out during non-international armed conflicts and those occurring outside such contexts. The study also sheds light on the different interpretations of the concept of “attack” within the context of the IHL and assesses the degree of protection afforded to civilians and their objects in light of the distinctive features of cyberspace.

**Results:**

The paper demonstrates that even though IHL provides a fundamental framework for such operations, its application to cyber operations is constrained by structural challenges, given the specificity of its infrastructure and the uncertainty surrounding civilian digital data. These challenges impede the practical application of the principles of distinction, proportionality, and precaution.

**Conclusions:**

The study concludes that the concept of “attack” needs to be reinterpreted considering the indirect harm inflicted on civilians resulting from cyber operations. It also manifests the need to raise the scope of legal protection encompassing fundamental civilian digital data, and confirms the possibility of developing a specialized international legal framework that governs cyber operations whether through the creation of an additional protocol or a treaty specific for such operations. Finally, the study further affirms the necessity to establish a neutral international mechanism that can conduct fact-finding tasks, investigate violations, and assign liabilities so as to promote better adherence to humanitarian principles in contemporary armed conflicts.

## Introduction

Against the backdrop of the accelerating information and communications technology revolution, access to cyberspace has become widely available, transforming the world into a closely interconnected network of data and communications. This shift has coincided with states’ movement toward digitization across all vital and essential sectors. Indeed, United Nations reports indicate that most countries now rely on information and communications technology to deliver public services, including the military sector ( United Nations, 2024; Khater et al., 2025).

In this regard, modern military systems now depend on information and communication technology as States have created dedicated cyber defense units for their military forces and established offensive and defensive cyber capabilities across their command systems. The electronic networks now serve two purposes in military operations because armed groups use them for their various activities which include recruitment and training and communication purposes ( Khater, 2024).

In this context, military applications of information technology now extend beyond management and coordination because these tools have become widely accessible. The military applications of information technology now include enemy movement tracking which occurs during both active combat and peacetime periods of conflict ( Bobenrieth & Watts, 2024). Cyber operations operate through diverse methods, including hacking into systems for the aim to collect, transfer, destroy, alter or encrypt data ( Qutieshat et al., 2026; Abou Adel et al., 2024). For example, cyberattacks on electricity distribution substations in Ukraine in December 2015 caused a power outage affecting approximately 225,000 users. On 24 February 2022, just hours before Russia’s invasion of Ukraine, cyberattacks targeted Viasat’s network, resulting in the disruption of thousands of modem devices used by military and governmental entities and in internet outages for hundreds of thousands of users across Europe ( NATO Cooperative Cyber Defence Centre of Excellence, n.d.). As a result, the effects of cyber military operations can spread to essential civilian services which people depend on such as electricity, water and medical treatment, thereby increasing the humanitarian impact of these operations ( El Baroudy et al., 2025; Qiqieh et al., 2025). In some cases, these operations may create serious damage to both civilians and their property which may reach dangerous levels matching the damage from traditional military operations ( Gisel & Olejnik, 2018; Al-Mulla et al., 2026; Al-Rai et al., 2024).

In light of these developments, the research gap lies in the inadequacy of the legal framework governing cyber operations. Although the international community has affirmed that States’ application of data technology is subject to IHL provisions, referring to “internationally recognized legal principles, including, as appropriate, the principles of humanity, necessity, proportionality, and distinction” ( UN, 2013; 2015, para. 28). Adaptation of IHL provisions to military acts carried out within the cyberspace context continues to pose complex legal challenges. In particular, this concerns both the scope of its applicability and the standards for applying it in an environment characterized by distinctive technical rather than purely physical features.

The study’s problem revolves around the degree of compliance of IHL to cyber military operations, obstacles to its application, and the extent to which it is necessary to reinterpret the provisions of this law to comply with the specific nature of cyberspace and to guarantee an effective protection for civilians and their objects. From this basis, the study’s central hypothesis asserts that the existing IHL provisions, despite their application, in principle, to cyber operations, face critical shortcomings that necessitate their reinterpretation or the introduction of new rules specific to the nature of this space.

The significance of this study lies in the fact that it sheds light on the ongoing jurisprudential debate concerning the possibility and the method of applying the IHL to this type of operations. Indeed, defining when it is necessary to apply this law to cyber operations is very important due to its significant legal and humanitarian implications, given the obligations it entails for the conflicting parties. These obligations constitute adherence to the IHL principles and assumption of responsibility for any potential violations, including war crimes. Therefore, this study may constitute a valuable scholarly contribution, as it helps in the restructuring and reinterpretation of the problematic aspects surrounding cyber military operations and in the development of laws regulating these operations under the provisions of the IHL.

## Methods

Through an inductive methodology, the research analyzes the International Humanitarian Laws regulating cyber military activity and assesses their applicability to cyber military operations. The researchers review international legal treaty and customary documents along with reports from specialized organizations, namely the International Committee of the Red Cross and relevant operational practices. This approach aims at evaluating the effectiveness and sufficiency of the existing legal framework, and determines the challenges impeding its application in the cyber space, most precisely the ambiguity surrounding the concept of “attack” and the scope of protection granted to civilians.

The study relies on an inductive analytical approach by analyzing the IHL regulations and assessing their applicability to cyber military operations. This analysis is based on an investigation of pertinent international legal texts—both treaty-based and customary—along with reports from specialized organizations, particularly the International Committee of the Red Cross, as well as related operational practice. This approach seeks to evaluate the adequacy of current legal framework and to identify the challenges that hinder its applicability to the cyber environment, especially the concept of “attack” and the scope of civilian protection.

The study focuses on laws governing cyber military operations, namely; the law of armed conflict (Jus in bello), with an emphasis on the conditions for this law’s applicability and its criteria, and an analysis of the challenges it faces when applied in cyberspace. By contrast, the study does not address the legality of cyber attacks from the perspective of “Jus ad bellum”, due to the different legal nature of these two bodies of law. International Humanitarian Law concerns the conduct of parties to armed conflicts and aims to protect their victims, whereas Jus ad bellum relates to determining the legality of states’ resort to the use of violence in international relations, in accordance with Article 2(4) of the United Nations Charter, which prohibits the use of force or the threat thereof, subject to its exceptions. Accordingly, the first body of law is linked to the existence of an actual armed conflict, while the second concerns the legality of resorting to force between states ( Khater, 2022b; Khater, 2022a).

## Results

The study demonstrates that applying the rules of IHL to cyber military operations is possible, in principle, however, this application remains conditional and still faces obstacles given the technical and structural characteristics of the cyberspace. The study affirms the applicability of IHL to cyber operations conducted in the context of an armed conflict, whether on the international or national level, provided that these operations are directly linked to violations acts. However, this rule remains questionable in the cyber environment, as it is not easy to differentiate between acts of a military nature and operations of a civilian nature. This will result in an uncertainty concerning the scope of suitability of the IHL in this context.

The study shows that the issue of attribution in cyber operations represents the main barrier which prevents the adequate application of the IHL because it remains impossible to identify operational controllers and link operations to particular states or entities. The study shows that attribution challenges in cyber operations represent the main barrier which prevents proper implementation of international humanitarian law because it remains impossible to identify the perpetrators and associate operations to particular States or entities. This may create a practical gap between the legal rules and the possibility of their practical enforcement. The study also highlights the complex legal concerns raised by the application of the said Law to acts conducted outside the context of kinetic hostilities, in light of the criteria of “use of armed force”, “organization”, and “intensity”, particularly given the immaterial nature of these operations and the possibility of their execution without direct physical interaction between the parties.

The study further shows that cyberspace’s characteristics constrain the adequate enforcement of the IHL provisions—particularly the principle of distinction, the ban of random attacks, and the rules of proportionality and precautions during attacks. This is due to the dual-use function of digital infrastructure together with the complete interconnection of civilian and military systems.

The study also affirm that a new legal system is developing to safeguard civilian digital data even though international bodies have not yet agreed on whether this data constitutes protected civilian objects. Nevertheless, the disruption or manipulation of this data may lead to serious humanitarian consequences comparable to—or even exceeding—the damage resulting from the destruction of physical objects, revealing a gap in the current scope of protection.

The study also emphasizes that traditional interpretations of the concept of “attack” do not apply to cyber operations due to the novelty and specificity of this kind of violations. Restricting the concept to direct material damage only leads to the exclusion of a wide range of operations which disable essential services in public facilities without causing actual physical destruction. Indeed, the concept of attack requires a functional definition which should be based on the resulting effects and outcomes of its implementation which include loss of operational capacity and civilian harm.

Accordingly, the research results demonstrate that the current legal framework which initially covered cyber operations continue to experience fundamental restrictions which diminish its operational capabilities. This may create actual shortcomings in safeguarding civilians and their belongings which require development of a specialized interpretative method to match the distinct requirements of this field.

## Discussion

### The scope of applicability of IHL to cyber military operations

Cyber operations are technological activities targeted at computer systems, networks, or internet-connected devices with the aim of using them for military needs ( Khater, 2023). Although IHL does not include specific treaty rules governing these operations, this does not mean that these operations are deemed legal. In this regard, the Martens Clause provides permanent protection to both civilians and combatants in situations that fall outside the specific regulations of the Geneva Conventions and their additional protocols and all other international treaties ( Additional Protocol 1, 1977, preamble). The International Court of Justice has confirmed that the text has effectively addressed the rapid progress of military technology ( ICJ, 1996).

Despite the fact that it is usually agreed upon that the IHL is enforceable, in principle, in the context of cyber space, practical application of this law reveals crucial gaps due to the intangible nature of these operations, as well as the complex interconnectivity of digital infrastructure. This will lead that some operations may fall within a “gray area” and be difficult to conceptualize, which requires investigation of the extent to which the general rules of IHL may apply to cyber operations (Smedes, 2025). It is also important to emphasize that the applicability of these rules does not, in any way, encourage or legitimize conflicts. In this regard, two different scenarios emerge; the first is when the cyber operation is carried out as a part of an armed conflict, and the second is when the act takes place independently from any kinetic military operation.

Recently, many armed conflicts have seen parties involved in both international and non-international fights use cyber operations to attack their enemies while they conduct actual military battles. Russia launched a major cyber attack on Viasat’s satellite network in Ukraine which started hours before its 2022 invasion because the attack caused modem and router outages throughout Ukraine and Europe. Russia made multiple efforts to interrupt Starlink service in Ukraine by conducting both cyber and physical attacks against the system. Similarly; these operations were used by both Israel and Hamas as a way of gaining superiority during the armed conflict from 2023 to 2025. The operations included hacking into websites belonging to IDF, Hamas, and other government agencies, in addition to disrupting communications systems throughout the Gaza Strip.

IHL applies to cyber operations carried out nationally or internationally, provided there is a close link to hostilities ( ICTY, 2002). Consequently, any act of warfare based on cyber technology must be subject to the IHL By adopting IHL treaties, States aim to regulate all conflicts and current and future acts of warfare ( Protocol I, 1977) The International Court of Justice endorsed this by noting that the established principles and rules of international humanitarian law apply “to all forms of warfare and to all types of weapons,” including “those of the future” ( ICJ, 1996, para. 86).

If cyber operations are conducted against a particular advisory to cause damage, such as by tampering with an air traffic control system resulting in the crash of a civilian aircraft-would be deemed a prohibited method of warefare under IHL ( ICRC, 2011). However, the issue becomes more complex when cyber operations are carried out independently of any armed conflict. While some consider that such operations include those conducted by a belligerent party against an adversary, others argue that they encompass operations executed to support the military effort of an attacking party. Under both interpretations, not every cyber operation executed during hostilities falls within the scope of IHL. For instance, operations carried out by individuals or private entities to obtain data from a competing company in an enemy state for the purpose of gaining a market advantage cannot be regarded as having been conducted in the context of an armed conflict. However, if a Ministry of Commerce in a given state conducts a cyber operation against a private company in an enemy state to obtain trade secrets during warefare, then—according to the first interpretation—IHL would apply, as the operation is carried out by a party involved in the hostilities against a company belonging to the advisory state. In contrast, under the second interpretation, IHL will not comply since the link between the cyber operation and the conflit is not sufficiently strong ( Schmitt, 2017).

In light of the shortcomings of both interpretations, reliance can be placed on the concept of the “nexus to hostilities”, developed by the International Committee of the Red Cross, which refers to an act “specifically designed to directly cause the required level of harm in support of one party to the conflict and to the detriment of another” ( Çetinkaya, 2016). Therefore, in terms of distinction, cyber operations that are designed to harm a party in an armed conflict for the benefit of another party should be part of that armed conflict, regardless of who carries out those cyber operations.

Although it is generally agreed upon that the IHL governs cyberspace military activities that take place during actual conflict situations, there remains uncertainty about which specific conditions determine whether those activities create an armed conflict according to IHL ( ICRC, 2023). To begin with, the 1949 Geneva Conventions replaced the term “war”, used in the Hague Conventions, with the notion of “armed conflict”. This change was intended to broaden the Conventions’ applicability scope and to render the enforcement of IHL contingent upon a factual, case-by-case review based on objective criteria ( Pictet, 2020). This raises the question of whether isolated cyber operations, detached from any kinetic military actions, can be characterized as an armed conflict under IHL. It should be emphasized that the purpose of determining the existence of an armed conflict is to establish the applicability of international humanitarian law; this assessment is legal in nature rather than political ( ICRC, 2023).

### Issues of adaptation and attribution of cyber military operations under international humanitarian law

International humanitarian law governs international and non-international armed conflicts with different legal rules. IHL treaties do not include a specific interpretation of international hostilities. Nonetheless, the Appeals Chamber of the International Criminal Tribunal for the former Yugoslavia held that hostilities exists “whenever there is a resort to armed force between States” ( ICTY, 1995, para. 70). Therefore, for an act to be deemed as an international armed conflict, two essential elements must be met: first, the conflict must take place between at least two States; and second, there must be a resort to armed force between them.

As for armed group, they have a “single person or group that plans and controls military operations without relying on higher ranks. In contrast, most uncoordinated hacking groups require state intervention to assign their cyber activities with a particular level of responsibility i.e., for a state to claim control over its cyber activities it must provide specific instructions regarding the nature, degree and duration of its attacks.

In any case, it is not sufficient, for the purpose of attributing cyber operations—whether committed by an organized armed group or by a group of unorganized individuals—to a state, and thereby classifying the act as part of hostilities, that the state’s role be limited to providing the software or technical equipment used, or that the operations be launched from its territory ( Schmitt, 2017).

For operations occurring within a State to be classified as non-international hostilities in accordance with Common Article 3 and customary IHL, it must meet the intensity criterion and involve at least two organized parties—namely, a state on the one hand and one or more organized armed groups on the other, or between such groups themselves ( Sassòli, 2024). In this respect, the ICC for the former Yugoslavia has developed indicative criteria that may serve as indicators of both the intensity of hostilities and the degree of organization of the parties. However, these factors are not exhaustive, and the assessment depends on a comprehensive evaluation of the various factual indicators specific to each case ( ICRC, 2020).

Concerning the element of organization, existence of an actual contact between members of an armed group, is not always necessary to be categorized as organized under IHL. A group may organize itself in the cyber space and carry out their operations without meeting physically. In this case, the group can meet the organization criterion, provided it has an organization leadership structure that determines its objectives, coordinates its operations, implement its rules and at the same time adheres to IHL fundamental provisions ( Hamdan, 2021). As for small unorganized operations conducted by individuals or a group of internet hackers, they do meet the organization requirement (Schmidt, 2017).

As for the element of intensity, the International Committee of the Red Cross has adopted a conservative position, as it deemed that cyber operations carried out outside the context of kinetic acts between a State and an organized armed group, or between these groups, are unlikely to reach the degree of the violence required to categorize under non-international armed conflict ( ICRC, 2023). Based on this, acts committed by nongovernmental parties and that result in damages and disruptions of important infrastructure, even though they may classify as cyber crimes, do not attain the degree of intensity to be categorized under non-international armed conflict.

In addition, some limited operations, such as hacking official websites, distributing riot calls, and stealing, modifying, or deleting data, are not sufficiently intense to qualify as non-international armed conflict, irrespective of the cyber nature of the operation. An instance of this would be the Russian minority calling for unrest in Estonia over cyberspace in 2007 ( ICRC, 2020; Schmitt, 2017). However, technological advancement cannot be ignored. Some cyber operations may lead to violent outcomes similar to those destructive damages resulting from kinetic attacks as they target critical infrastructure such as dams, air navigation systems and power plants. This level of intensity allows for their classification as non-international armed conflict despite the absence of standard kinetic military operations ( Droege, 2012).

For this reason, reconciling cyberspace with the legal system that governs armed conflict has created a range of challenges. Among them are the difficulties in determining whether or not IHL applies to a cyber operation carried out without any kinetic hostilities; difficulty in determining attribution; and challenges in the application of rules protecting civilians and their objects. In addition, these issues are further compounded by the dual-use nature of the cyberspace and its networks (for military and civilian purposes at once), and the ongoing debate about the extent to which civilian digital data and objects are protected.

### Reinterpreting the concept of “Attack” in cyber operations

The growing use of digital technology by civilian infrastructure which supports vital services for the public needs immediate protective measures against cyber operations and any damage that might occur during such operations to safeguard both civilians and their property. International humanitarian law grants specific protection to designated civilian infrastructure during both international and non-international armed conflicts which prohibits attacks on these protected areas through conventional military methods and cyber warfare. This protection covers medical facilities (whether permanent or temporary), hospitals, blood transfusion centers, preventive medicine facilities, medical warehouses and pharmacies ( Protocol I, 1977). The protection also extends to vital resources including drinking water and agricultural irrigation systems. Therefore, the protection covered by IHL is contingent upon whether, or not, a cyber operation is deemed an “attack” under this law.

In this regard, “attacks” are viewed as “acts of violence against the adversary, whether offensive or defensive”. The term “acts of violence” do not only encompass acts involving the use of physical force, as the use of biological or chemical weapons is also considered an attack, even though it does not necessarily involve direct physical force ( Protocol I, 1977). Accordingly, the criterion for characterizing an operation as an attack is not linked to the nature of the means used, but rather to the violent consequences resulting from it ( Schmitt, 2017).

On this basis, cyber operations that cause physical injury to persons or material damage to property—excluding computer programs or data—may be regarded as “acts of violence”, that is, “attacks” within the meaning of international humanitarian law. Any cyber operation that can reasonably be expected to cause death, bodily injury, or material damage is considered an “attack”, whether these violent consequences are direct or indirect effects of the operation. For example, a cyberattack targeting an electrical power grid may result in a power outage in hospitals, leading to the death of patients in intensive care units. Similarly, cyber operations may target dams, causing the release of water and the destruction of vast areas of farmland, villages, and nearby cities. This interpretation of the concept of “attacks” is consistent with the nature and purpose of IHL, which is a result-oriented body of law aimed at protecting civilians from the effects of armed conflict to the greatest extent possible.

In contrast, cyber operations that are devoid of any form of violence are not considered attacks, such as cyber espionage and intelligence gathering, psychological warfare operations, or interference with wireless communications or television broadcasting ( ICRC, 2024). These operations do not rise to the level of military operations and, as such, are not in themselves subject to the provisions of international humanitarian law.

However, limiting the characterization of cyber military operations as “violent consequences” may lead to excluding an important category of cyber operations—namely those aimed at disrupting civilian infrastructure without necessarily destroying it, such as disabling electricity grid control systems, communications networks, or banking systems, thereby depriving them of functionality without causing direct physical damage ( Biggio, 2025; Bobenrieth & Watts, 2024).

This interpretation is supported by the definition of military objectives in Additional Protocol I, which considers them to be objects that make an effective contribution to military action and whose “partial or total destruction, capture or neutralization, in the circumstances ruling at the time, offers a definite military advantage” ( Protocol I, 1977). This indicates that “neutralization” or “disruption” itself can be the object of an attack.

Moreover, restricting the notion of “attack” to cyber operations that only produce physical violent consequences is inconsistent with the purpose and objective of the provisions regulating hostilities, which is to afford protection of civilians and civilian objects from the effects of such acts. This narrow interpretation would exclude from the scope of the fundamental principles of international humanitarian law those cyber operations that are intended to, or are expected to, “disrupt” civilian infrastructure, even though the potential effects of such operations on civilians and civilian objects may be severe. Excluding them from the protective scope of international humanitarian law would therefore be contrary to the essence of the principle prohibiting direct attacks against civilians and civilian objects ( ICRC, 2024).

In this context, an important question arises regarding the threshold at which loss of functionality resulting from the disruption of civilian infrastructure is sufficient to classify a cyber hostile operation as an attack. Experts involved in the Tallinn Manual have expressed differing views on this issue. One group considers that such classification should be limited to situations in which the physical components of the targeted cyber infrastructure require repair or replacement. Another group holds that it should also include cases where restoring functionality requires reinstalling the operating system or the data on which the system relies to perform its intended functions. A third group argues that the manner in which the loss of functionality occurs is not decisive; rather, the key criterion is that the system no longer operates as it was designed to do ( Schmitt, 2017).

Researchers agree with Sassòli (2024) that the criterion for determining whether a cyber operation qualifies as an attack lies in its effects on civilian infrastructure, regardless of its technical nature or the means required for its subsequent repair. Just as the assessment of whether a kinetic military operation targeting a state’s telecommunications control center constitutes an “attack” under international humanitarian law does not depend on how the center is repaired or how its functions are restored, a cyber operation targeting such a network should likewise be classified as an attack based on the consequences of its disruption.

The question therefore remains open regarding the minimum level of harm or disruption required to characterize a cyber operation that causes such effects as an “attack”. This is particularly the case given differences among states in how they define essential civilian needs, which means that the answer to this question is not fully resolved.

In short, even if an operation is not identified as an ‘attack’, the fundamentals of International Humanitarian Law still apply. There are rules of the same law that exist outside of what is defined as an ‘attack’ ( Droege, 2012). The central distinction is between ‘operations’ and ‘attacks’ i.e. other types of actions. International Humanitarian Law also provides civilians with ‘general protection against the dangers arising from military operations’ ( Additional Protocol 1; 1977) and obligates military forces to exercise ‘due care’ to avoid harming civilians and civilian objects when conducting military operations ( Dinniss, 2012). Therefore, a cyber operation that would not be considered an ‘attack,’ may fall under International Humanitarian Law if that operation is executed during a period of armed conflict ( ICRC, 2024).

### Limits on the application of international humanitarian law principles in protecting civilians in cyberspace

If IHL applies to a particular cyber military operation, then the “principle of distinction between military objectives and civilian objects” requires that cyber attacks not be carried out against purely civilian cyber infrastructure. However, this obligation faces significant practical difficulties in cyberspace, due to the close structural interconnection between civilian and military networks. Most military networks rely on civilian cyber infrastructure, such as undersea fiber-optic cables and satellites, as well as routing and communication devices. Conversely, land and maritime transportation systems and civil aviation traffic-monitoring systems are equipped with GPS navigation systems based on satellites, which are also used by the armed forces. As a result, distinguishing between purely civilian cyber infrastructure and purely military cyber infrastructure becomes exceedingly difficult.

The danger of this interconnection lies in the possibility that the effects of cyber operations may extend beyond the intended military objective. For instance, if a virulent virus—or a set of viruses—is deployed to target a hostile state’s military network, that malware may spread into its civilian networks and go beyond their boundaries, causing the disruption or destruction of civilian infrastructure that depends on those networks ( Droege, 2012). In such a case, the operation may be considered an indiscriminate attack under international humanitarian law, because it becomes impossible to direct it toward a specific military objective. As a result, it would qualify as a means or method of warfare that cannot be limited in its effects and is therefore prohibited under international humanitarian law in both international and non-international armed conflicts ( Additional Protocol I, 1977).

Moreover, according to the traditional interpretation of the concept of military objective, dual-use objects—i.e., those used for both civilian and military purposes—are lawful military objectives if they make an effective contribution to military action, whether by their nature, location, purpose, or use, and if their total or partial destruction, capture, or neutralization provides a definite military advantage ( Additional Protocol I, 1977). However, the strict application of this concept could lead to characterizing many objects that constitute an essential part of the civilian and military infrastructure of cyberspace—or potentially cyberspace as a whole—as military objectives ( Schmitt, 2017; Sassòli, 2024). Under this approach, such objects would not be afforded protection against direct attacks, whether cyber or kinetic ( Additional Protocol I, 1977), despite the fact that this may result in the interruption of civilian use of that space. Given states’ growing reliance on cyberspace to meet the vital basic needs of civilian populations—for food, water, energy, health services, financial services, and others—the humanitarian consequences arising from this interpretation are likely to be a matter of serious concern ( Gisel & Olejnik, 2018).

In any event, even if certain parts of cyberspace infrastructure upon which essential civilian services depend become military objectives due to dual use, any attack against them remains governed by the principles of proportionality and precautions in attack. To apply these principles, it is necessary to assess the expected incidental civilian harm of any military operation ( Additional Protocol I, 1977). Given cyberspace’s dual-use nature and the interconnection between civilian and military networks, the assessment of incidental harm must take into account the direct and indirect expected effects of cyber attacks—whether they are physical damage or the impairment of civilian infrastructure’s ability to perform its functions even in the absence of physical damage ( Droege, 2012). This includes undermining civilians’ access to their basic vital needs in societies that increasingly rely on digital services and data. This raises doubts about whether any cyber operation would be consistent with the principle of proportionality in light of the potential effects compared with the certain and concrete military advantage it may yield. Moreover, the interconnected nature of networks makes it difficult to predict all the impacts that cyber military operations may cause, which further complicates the application of the principle of proportionality ( Sassòli, 2024).

On the other hand, it is often argued that cyber military operations are highly precise; therefore, they can be directed at specific targets, which may reduce incidental harm to civilians and civilian objects resulting from the attack ( Droege, 2012). However, cyberspace’s dual-use nature makes it particularly difficult to comply with the principle of precautions in attack, which requires the attacker to take all feasible measures to verify that the objective to be attacked is a military objective ( Additional Protocol I, 1977). In addition, the complexity of these operations, the high likelihood that they will affect civilian networks, and limited understanding of their nature and their outcomes by those responsible for their management make it necessary—whenever possible—to rely on technical experts to assess whether appropriate precautionary measures have been taken ( Schmitt, 2017). Such measures should be incorporated in advance into the design of cyber operations, given the automated nature of most of them ( Sassòli, 2024). Indeed, many defensive cyber operations are programmed to target the source of the attack without human intervention, which may undermine the principles of proportionality and precautions by failing to ensure that the defensive action is proportionate to the hostile action, or by causing losses that exceed what is necessary to repel the attack. The effects may even extend to targeting civilian computer systems without distinguishing their civilian or military nature ( Droege, 2012).

In addition, the principle of precautions against the effects of attacks also requires that the parties to a conflict refrain from placing military objectives within or near densely populated areas, and that civilians and civilian objects be kept away from the vicinity of military objectives ( Additional Protocol I, 1977). These “negative” precautions, according to the Tallinn Manual, include: separating civilian cyber infrastructure from military infrastructure; separating computer systems on which essential civilian infrastructure depends from the Internet; protecting essential civilian data; making advance arrangements to ensure the timely repair of important computer systems; digitally recording significant cultural or spiritual objects to facilitate reconstruction if they are destroyed; and using anti-virus measures to protect civilian systems that may be damaged or destroyed during an attack on military cyber infrastructure ( Schmitt, 2017).

In practice, these measures are rarely implemented by states for financial reasons and in order to ensure network flexibility ( Sassòli, 2024). Moreover, the flexibility of cyberspace allows users to reroute data flows immediately. If we assume that a state’s armed forces use a civilian social-media network to transmit military information, it becomes difficult to determine which part of that network is being used in particular, and they can easily move to another network ( Schmitt, 2017). This flexibility raises doubts about whether the destruction or disruption of a dual-use objective in such circumstances would result in a “definite military advantage,” as required by the second element of the definition of a military objective.

On the other hand, as states increasingly adopt e-government and rely more heavily on information technology and the digitization of essential civilian data to meet civilians’ vital needs, the question arises as to whether such data enjoys protection as civilian objects under IHL Because some civilian data concerns specific civilian material objects that enjoy special protection, this protection should extend to relevant related data. The obligation to respect and protect medical facilities and units is presumed to include the protection of specific medical data relating to those facilities ( ICRC, 2023), such as patient records in hospitals, data held by blood-transfusion centers, vaccine data, and data held by humanitarian organizations engaged in relief operations. To protect the digital components of protected civilian entities and objects that are entitled to the distinctive emblem under international humanitarian law—especially medical and humanitarian facilities—the ICRC proposed adopting a digital emblem to distinguish these components during armed conflicts, with the aim of protecting them from cyber attacks ( ICRC, 2022).

The challenge arises with civilian data that does not fall within the scope of special protection—for instance, social security data, tax records, bank accounts, election records—and whether it is covered by the general protection afforded to civilian objects under the rules of international humanitarian law during the conduct of hostilities. State practice provides no clear answer to this issue, which also remains debated among experts ( Gisel et al., 2020; Al-Rai et al., 2026a).

The deletion or tampering with such essential civilian digital data may lead to rapid and complete paralysis of the vital services on which civilians depend, potentially causing harm comparable to—and possibly exceeding—that caused by the destruction of traditional material civilian objects. It is also not reasonable to exclude civilian data from the protection of civilian objects merely because it is stored digitally rather than in paper records ( ICRC, 2023; Al-Rai et al., 2026b).

Arguing that the deletion or tampering of such essential civilian digital data is not prohibited under IHL in today’s world—largely dependent on digital technology—either because it would not amount to an “attack” under that law or because this data would not constitute an object prohibited from being attacked, is difficult to reconcile with the purpose and objective of international humanitarian law. That purpose is to balance States’ interests in conducting military operations effectively against the suffering that those operations cause to both civilians and combatants ( Schmitt, 2019). Excluding essential civilian digital data from the protection IHL provides for civilian objects would create a major protection gap. To bridge this gap, essential civilian digital data must be included in protection in any amendment to the Fourth Geneva Convention. Until this happens—along with the opposition it may face from states and their differing views on what qualifies as “essential” digital data—states should adopt humanitarian policies aligned with the purpose of international humanitarian law. This can be done by granting special protection to certain “essential civilian functions or services”, so that States undertake to refrain from cyber operations against civilian material or digital infrastructure if such operations would disrupt these services. States should also refrain from cyber operations that fall outside the rules specifically governing attacks when the expected negative effects on civilians are excessive in relation to the concrete and anticipated military advantage.

## Conclusion

This study uncovers the potential, in principle, for applying international humanitarian law to cyber military operations, yet it also demonstrates the existence of structural limitations that restrict its practical effectiveness. It further shows that traditional interpretations of central concepts in IHL—especially the notion of “attack” as well as the principles of distinction and proportionality—do not adequately capture the technical and non-material characteristics of cyber operations. This is particularly true in light of the dual-use nature of digital infrastructure and the structural interconnection between civilian and military systems, which reduces the actual scope of protection afforded to civilians and civilian objects and creates clear legal gaps. The study also highlights the problem of protecting civilian digital data in the absence of international consensus on whether it qualifies as a “material object” within the meaning of international humanitarian law, despite the potentially severe humanitarian consequences resulting from its disruption or tampering, comparable to or even exceeding traditional material harms.

Accordingly, the study concludes that it is necessary to reinterpret the concept of “attack” in a functional manner, such that it includes not only physical destruction, but also loss of functionality and indirect effects that affect civilians as a result of cyber operations. It also calls for expanding the scope of legal protection to include essential civilian digital data—especially that related to vital infrastructure and public services. In addition, it recommends developing a specialized international legal framework governing military operations in cyberspace that takes into account the specific nature of these operations—whether by adopting a separate international convention or an additional protocol to the four Geneva Conventions. Such a framework should contain clear rules defining cyber attacks, protecting civilian digital data, and strengthening attribution and accountability mechanisms. Finally, the study recommends establishing international oversight mechanisms to ensure respect for humanitarian principles by creating a neutral, permanent international commission to conduct fact-finding regarding cyber operations suspected of causing severe violations of IHL, and to provide technical and legal reports on that matter.

Lastly, the study emphasizes strengthening international cooperation and establishing shared technical rules and procedures that help trace the source of cyber operations and analyze their humanitarian impacts, thereby enabling more accurate identification of the responsible party and supporting the more effective application of international humanitarian law in cyberspace.

## Ethical considerations

Not applicable.

## Data Availability

No underlying data are associated with this article. This study is based on doctrinal and analytical legal research relying on international treaties, customary international humanitarian law, legal literature, and reports from specialized organizations. Therefore, no datasets were generated or analyzed during the current study. All sources used in this manuscript are publicly available and are cited in the references section.
