# Supplementary material for: Cyber Military Operations under International Humanitarian Law: Interpreting the Concept of “Attack” and Challenges in Protecting Civilians
Source: F1000Res. 2026 Jul 9;15:919. Originally published 2026 Jun 12. [Version 2] doi: 10.12688/f1000research.182654.2 (PMC13316166; doi:10.12688/f1000research.182654.2)
Supplement: Peer review history

# Reviewer report: Reviewer response for version 2

**DOI:** 10.5256/f1000research.205222.r499875

**Published:** 2026-07-10

**Authors:** Mohamad Hidayat Muhtar

**Affiliations:** 1 Universitas Negeri Gorontalo, Gorontalo, Indonesia

Revision has been done well and in accordance with our request.

Is the work clearly and accurately presented and does it cite the current literature?

Partly

If applicable, is the statistical analysis and its interpretation appropriate?

Not applicable

Are all the source data underlying the results available to ensure full reproducibility?

No source data required

Is the study design appropriate and is the work technically sound?

Yes

Are the conclusions drawn adequately supported by the results?

Yes

Are sufficient details of methods and analysis provided to allow replication by others?

Partly

Reviewer Expertise:

Law

I confirm that I have read this submission and believe that I have an appropriate level of expertise to confirm that it is of an acceptable scientific standard.

# Reviewer report: Reviewer response for version 1

**DOI:** 10.5256/f1000research.201616.r494118

**Published:** 2026-06-29

**Authors:** Hamzeh Abu Issa

**Affiliations:** 1 Applied Science Private University, Amman, Jordan

The article addresses an important and timely issue: the application of international humanitarian law to cyber military operations, particularly the interpretation of “attack” and the protection of civilians and civilian objects in cyberspace. Its main argument is that IHL applies in principle to cyber operations during armed conflict, but its practical application is limited by attribution difficulties, dual-use infrastructure, interconnection between civilian and military systems, and uncertainty regarding the protection of civilian digital data.

 The article is generally relevant and technically sound. It engages with appropriate legal sources and current literature, including ICRC materials, the Tallinn Manual, and leading scholarship. The doctrinal and analytical approach is suitable, and the distinction between jus in bello and jus ad bellum is helpful.

Is the work clearly and accurately presented and does it cite the current literature?

Yes

If applicable, is the statistical analysis and its interpretation appropriate?

Not applicable

Are all the source data underlying the results available to ensure full reproducibility?

No source data required

Is the study design appropriate and is the work technically sound?

Yes

Are the conclusions drawn adequately supported by the results?

Yes

Are sufficient details of methods and analysis provided to allow replication by others?

Yes

Reviewer Expertise:

Criminal law

I confirm that I have read this submission and believe that I have an appropriate level of expertise to confirm that it is of an acceptable scientific standard.

# Response

**Published:** 2026-06-29

**Authors:** Maya Khater

The authors sincerely thank the reviewer for the positive evaluation of our manuscript and for the constructive comments.

 They greatly appreciate the recommendation for accepting the manuscript.

# Reviewer report: Reviewer response for version 1

**DOI:** 10.5256/f1000research.201616.r494122

**Published:** 2026-06-22

**Authors:** Mohamad Hidayat Muhtar

**Affiliations:** 1 Universitas Negeri Gorontalo, Gorontalo, Indonesia

This article examines the application of international humanitarian law to cyber military operations, with particular attention to the interpretation of the concept of “attack” and the challenges of protecting civilians and civilian objects in cyberspace. The article argues that IHL applies in principle to cyber operations conducted in the context of armed conflict, but that its practical application is limited by the specific features of cyberspace, including attribution difficulties, dual-use infrastructure, interconnection between military and civilian networks, and uncertainty regarding the legal protection of civilian digital data. The article’s central contribution lies in its argument that the concept of “attack” should be interpreted functionally, so that it can cover not only physical destruction but also loss of functionality and serious indirect effects on civilians.

 Overall, the article addresses an important and timely topic. It engages with a relevant body of literature, including ICRC materials, the Tallinn Manual 2.0, writings by Schmitt, Droege, Sassòli, Gisel and others, and more recent literature from 2024–2026. The discussion of civilian digital data, dual-use cyber infrastructure, proportionality, distinction, precautions, and attribution is relevant and useful. The article is generally clear in structure, moving from the applicability of IHL to cyber operations, to attribution, the concept of attack, and the protection of civilians. The conclusions are broadly consistent with the discussion.

 However, I would assess the clarity and presentation as only partly satisfactory. Some sections contain repetition, especially in the methods and results sections, and several arguments would benefit from tighter drafting and clearer legal framing. For example, the methods section repeats similar statements about the inductive and analytical approach. The results section also occasionally repeats points already made in the introduction and discussion. The authors should revise the manuscript to reduce repetition and improve coherence. Some terminology should also be corrected or standardized, such as “cyber space/cyberspace,” “attack/cyberattack,” “advisory/adversary,” “warefare/warfare,” and “conflit/conflict.” In addition, several sentences require language editing to improve readability and precision.

 The study design is appropriate for a doctrinal legal article. The use of an inductive and analytical method based on treaty law, customary IHL, ICRC reports, judicial decisions, and academic literature is suitable for the subject matter. The article does not require empirical or statistical analysis. The technical legal discussion is generally sound, especially in relation to the applicability of IHL, the threshold of armed conflict, distinction, proportionality, precautions, and the loss-of-functionality debate. The article also appropriately separates jus in bello from jus ad bellum, which helps clarify the scope of the study.

 That said, the methods and analysis are not yet described in sufficient detail to allow full replication by other researchers. For doctrinal legal research, “replication” does not require datasets, but it does require a transparent explanation of how legal sources were selected and analyzed. The authors should explain more clearly which categories of sources were reviewed, why these sources were chosen, and how the analysis was conducted. For example, the article could specify whether the authors prioritized primary legal sources, ICRC materials, state practice, academic commentary, or case law, and whether the analysis followed particular themes such as applicability, attribution, attack, distinction, proportionality, precautions, and civilian data. This would strengthen methodological transparency without requiring empirical data.

 The statistical analysis question is not applicable. The article is a doctrinal and analytical legal study and does not use statistical methods, empirical datasets, or quantitative analysis.

 Regarding source data, no source data are required. The article states that no datasets were generated or analyzed and that the study relies on publicly available legal materials, literature, and reports. This is acceptable for a doctrinal legal article.

 The conclusions are generally supported by the discussion, but I would assess this as partly rather than fully satisfactory. The article provides a reasonable basis for concluding that IHL applies in principle to cyber military operations and that there are important gaps concerning the concept of “attack,” attribution, dual-use infrastructure, and civilian digital data. However, some recommendations are ambitious and need stronger justification. In particular, the proposal to develop a separate international convention or additional protocol, and the recommendation to establish a neutral permanent international fact-finding mechanism for cyber operations, should be supported with more detailed reasoning. The authors should explain why existing mechanisms are insufficient, what form the proposed mechanism might take, how it would operate, and what political or legal obstacles may arise. The article should also distinguish more clearly between lex lata, meaning the current law, and lex ferenda, meaning proposed future legal development.

 The main points that should be addressed to make the article scientifically sound are as follows. First, the authors should revise the manuscript for language, consistency, and repetition. Second, the methodology should be made more transparent by explaining the selection and use of legal sources. Third, the authors should clarify the distinction between existing legal obligations and proposed reforms. Fourth, the recommendations concerning a new treaty, additional protocol, or international fact-finding mechanism should be developed in more detail. Fifth, the discussion of civilian digital data should more clearly explain the competing legal positions and the extent to which there is or is not international consensus. Finally, the article would benefit from a more explicit engagement with state practice and opinio juris, where available, especially on cyber operations, loss of functionality, and protection of data.

 In conclusion, the article makes a useful contribution to the literature on cyber operations and IHL. Its topic is current, its doctrinal approach is appropriate, and its main argument is relevant. I recommend acceptance subject to revisions, particularly improvements to clarity, methodological transparency, and the legal support for the article’s more ambitious reform proposals.

Is the work clearly and accurately presented and does it cite the current literature?

Partly

If applicable, is the statistical analysis and its interpretation appropriate?

Not applicable

Are all the source data underlying the results available to ensure full reproducibility?

No source data required

Is the study design appropriate and is the work technically sound?

Yes

Are the conclusions drawn adequately supported by the results?

Yes

Are sufficient details of methods and analysis provided to allow replication by others?

Partly

Reviewer Expertise:

Law

I confirm that I have read this submission and believe that I have an appropriate level of expertise to confirm that it is of an acceptable scientific standard, however I have significant reservations, as outlined above.

# Response

**Published:** 2026-06-29

**Authors:** Maya Khater

The authors sincerely thank the reviewer for the constructive comments and valuable suggestions, which have significantly improved the quality of the manuscript. We have carefully considered all of the reviewer's recommendations and addressed them in the revised version.

 Specifically, the methodology section has been expanded to provide a clearer explanation of the inductive and analytical legal approach, including the selection and analysis of primary and secondary legal sources, the analytical framework employed, and the thematic organization of the study. The manuscript has also been revised to improve its overall structure and readability, and to ensure consistency in legal terminology throughout the text.

# Reviewer report: Reviewer response for version 1

**DOI:** 10.5256/f1000research.201616.r494124

**Published:** 2026-06-19

**Authors:** Abdullah M Ehjelah

**Affiliations:** 1 Yarmouk University, Irbid, Irbid Governorate, Jordan

This paper represents a distinguished contribution to the growing body of scholarship addressing cyber military operations and the extent to which International Humanitarian Law (IHL) applies to them. The authors have successfully highlighted the legal complexities arising from the rapid evolution of cyberspace and its impact on contemporary armed conflicts, demonstrating a profound understanding of one of the most significant and challenging issues in modern international law.

 The study is characterized by a rigorous and well-structured methodology based on analytical and inductive approaches. The authors draw upon a wide range of sources, including both treaty-based and customary rules of International Humanitarian Law, as well as reports issued by specialized organizations. Their analysis reflects a strong ability to examine the legal framework governing cyber operations and to assess the applicability of IHL in this context. Furthermore, the paper provides a balanced and comprehensive discussion of the various interpretations of the concept of “attack” within cyberspace.

 The findings demonstrate the authors’ thorough understanding of the practical challenges associated with applying the principles of distinction, proportionality, and precaution to cyber operations. In particular, the study effectively explains the legal and technical difficulties arising from the unique nature of cyber infrastructure and the uncertainty surrounding civilian digital data. These observations provide valuable insights into the limitations of the current legal framework and the areas that require further development.

 The conclusion is particularly noteworthy for its innovative perspective and well-reasoned recommendations. The authors persuasively argue for a reconsideration of the concept of “attack” to encompass the indirect harm inflicted upon civilians through cyber operations. They also make a compelling case for extending legal protection to essential civilian digital data and offer a forward-looking vision for the development of a specialized international legal framework governing cyber operations, whether through an additional protocol or a dedicated international treaty. Moreover, the recommendation to establish a neutral international mechanism for fact-finding, investigation of violations, and attribution of responsibility constitutes a valuable and constructive contribution to ongoing discussions in this field.

 This study addresses the topic of the criminal protection of digital evidence in matters of criminal and civil law. It does so by explaining the criminal procedures that must be observed to protect this evidence and detailing the legal provisions that criminalize and penalize acts of assault on digital evidence in comparative laws. The importance of this study lies in the fact that countries worldwide are currently moving toward the use of information technology in various fields. Information technology is now widely employed in contract execution

 Overall, this manuscript makes a significant and timely contribution to the legal literature on cyber warfare and International Humanitarian Law. It reflects substantial scholarly effort, sound legal analysis, and a high level of academic quality. In my view, the article represents a valuable addition to the field and is worthy of indexation.

Is the work clearly and accurately presented and does it cite the current literature?

Yes

If applicable, is the statistical analysis and its interpretation appropriate?

I cannot comment. A qualified statistician is required.

Are all the source data underlying the results available to ensure full reproducibility?

Yes

Is the study design appropriate and is the work technically sound?

Yes

Are the conclusions drawn adequately supported by the results?

Yes

Are sufficient details of methods and analysis provided to allow replication by others?

Yes

Reviewer Expertise:

NA

I confirm that I have read this submission and believe that I have an appropriate level of expertise to confirm that it is of an acceptable scientific standard.

# Response

**Published:** 2026-06-19

**Authors:** Maya Khater

Dear Dr. Abdullah,

 Thank you for your valuable review and positive feedback. We greatly appreciate your time, expertise, and encouraging assessment of our work.

 Kind regards,

 Maya Khater
